# Supplementary figures and images for: USP27X variants underlying X-linked intellectual disability disrupt protein function via distinct mechanisms
Source: Life Sci Alliance. 2024 Jan 5;7(3):e202302258. doi: 10.26508/lsa.202302258 (PMC10770416; doi:10.26508/lsa.202302258)

Figure 2B

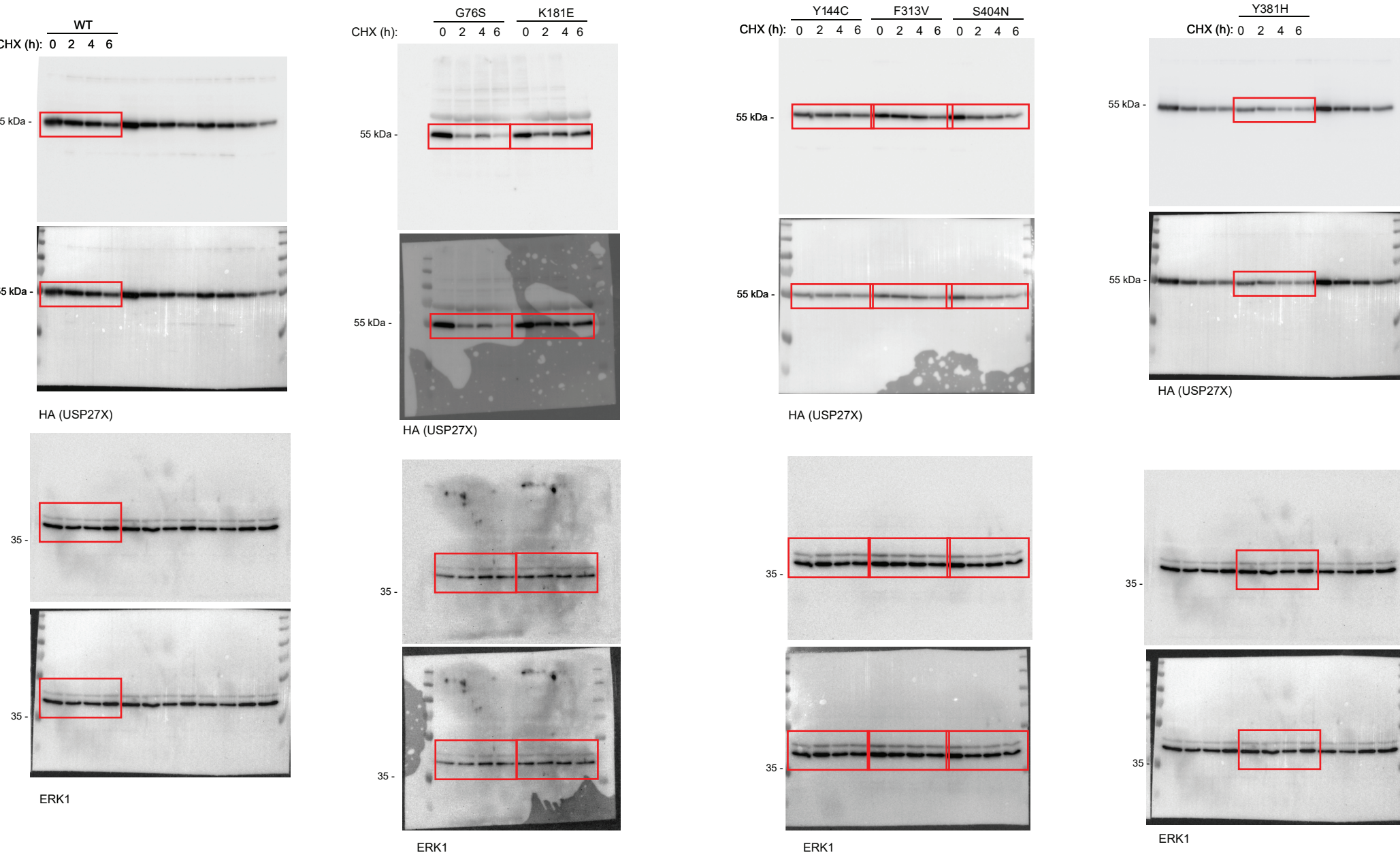

Supplement: Supplementary file 4 [file LSA-2023-02258_SdataF2.pdf]

Figure S3A

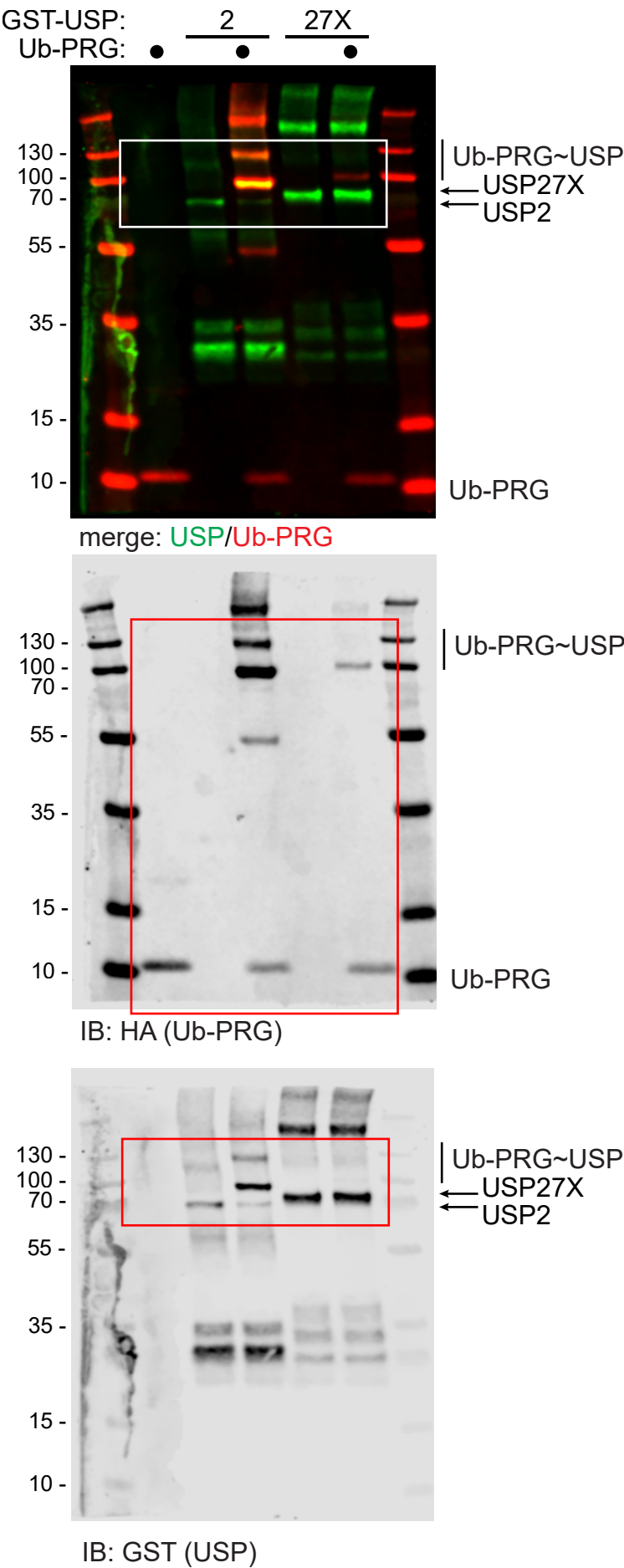

Supplement: Supplementary file 5 [file LSA-2023-02258_SdataFS3.1.pdf]

Figure S3B

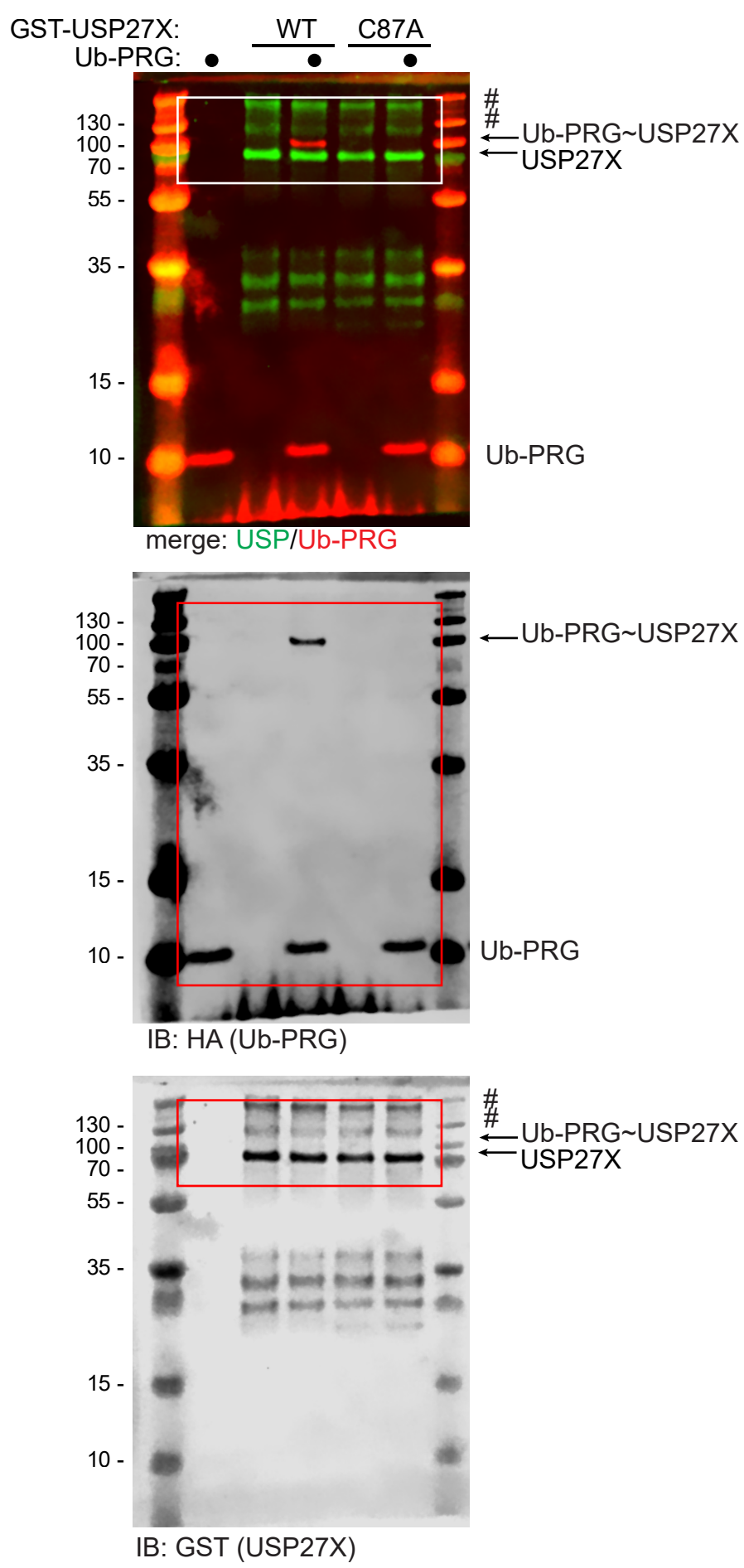

Supplement: Supplementary file 6 [file LSA-2023-02258_SdataFS3.2.pdf]

Figure S3C

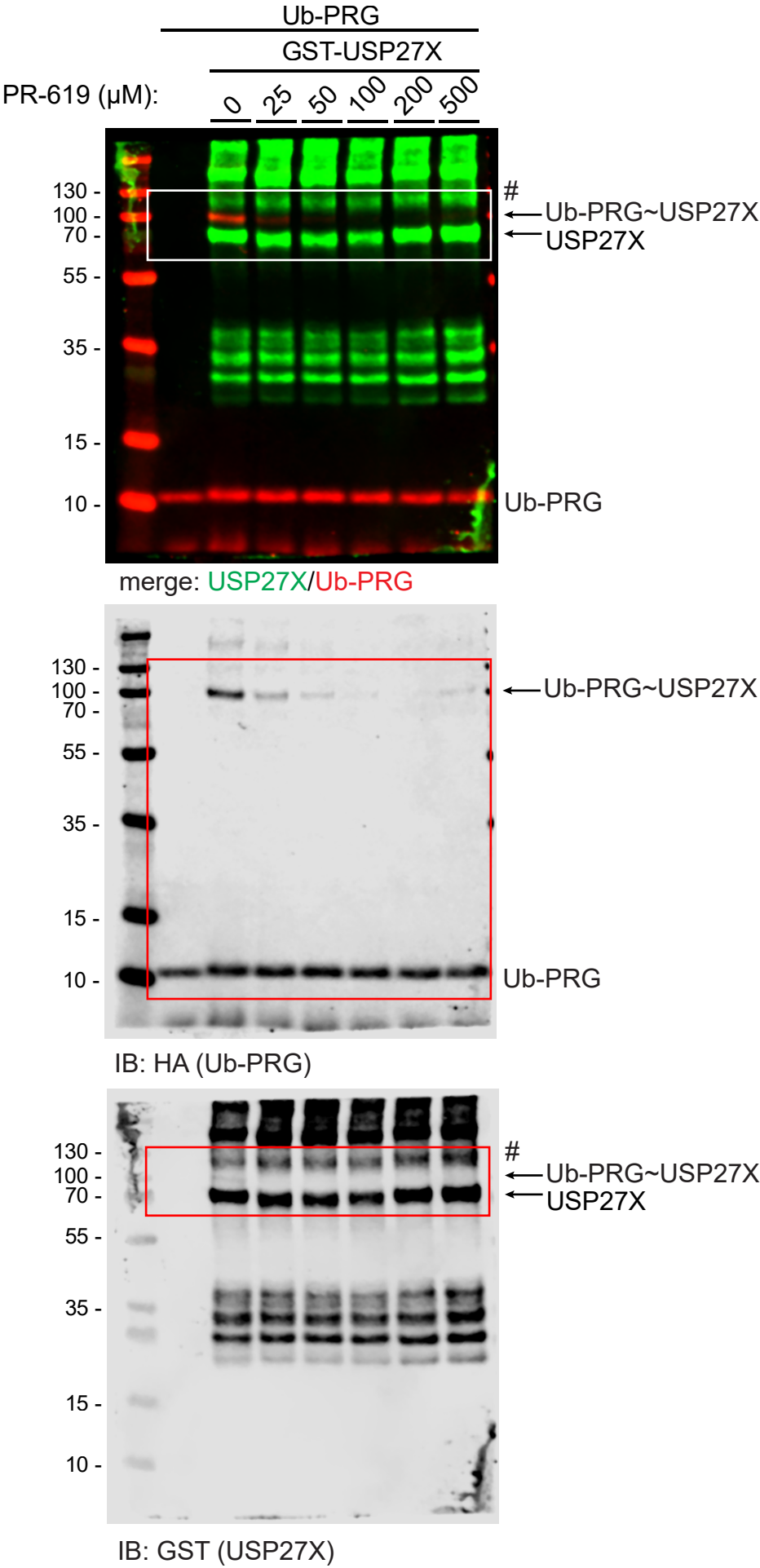

Supplement: Supplementary file 7 [file LSA-2023-02258_SdataFS3.3.pdf]

Figure 4A

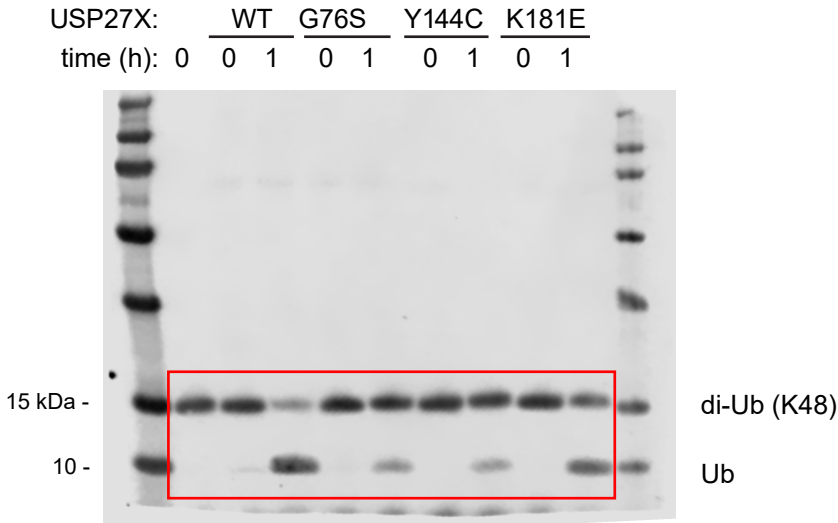

IB: Ubiquitin

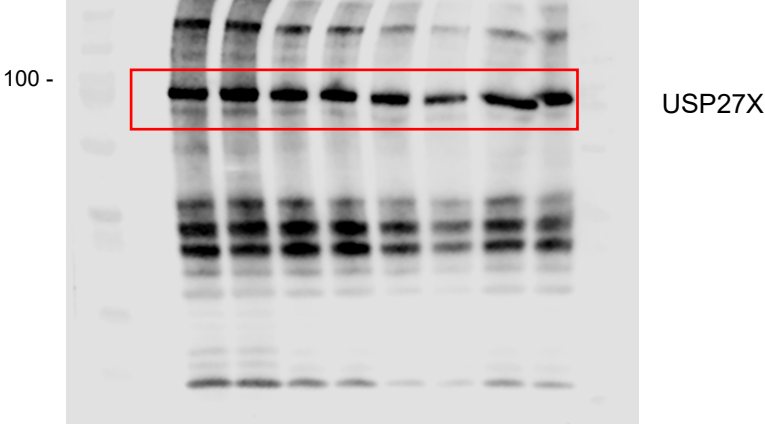

IB: GST (USP27X)

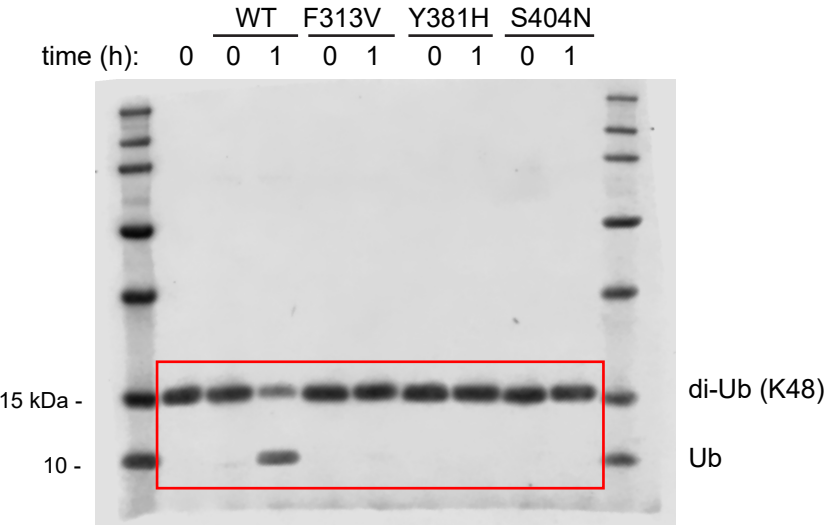

IB: Ubiquitin

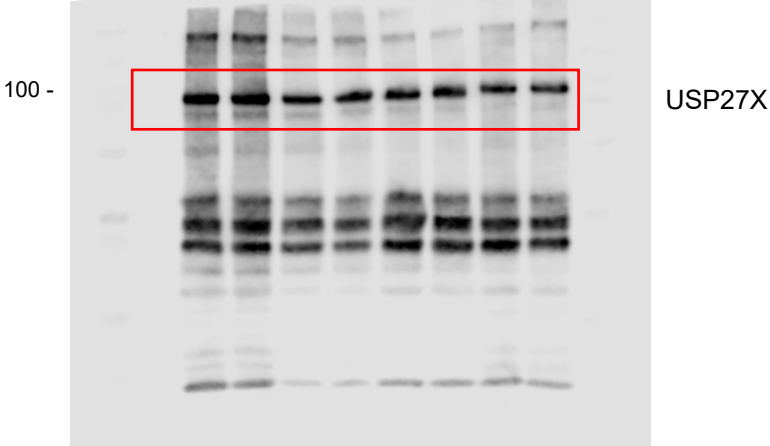

IB: GST (USP27X)

Supplement: Supplementary file 8 [file LSA-2023-02258_SdataF4.1.pdf]

Figure 4B

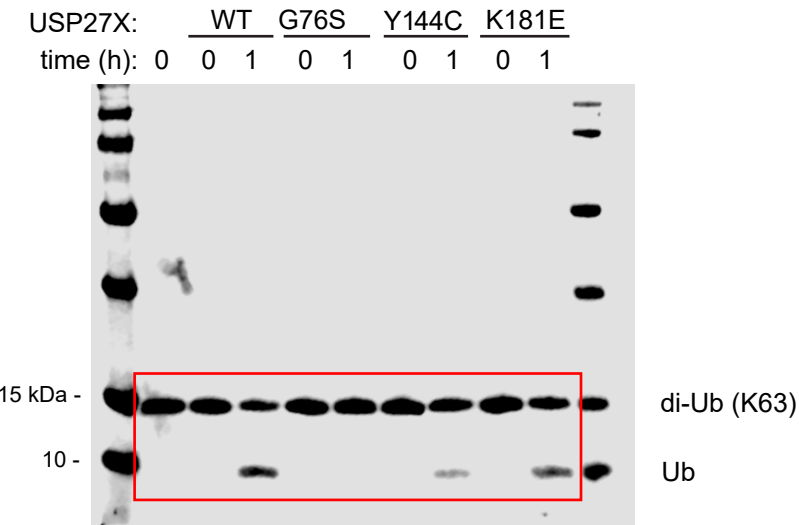

IB: Ubiquitin

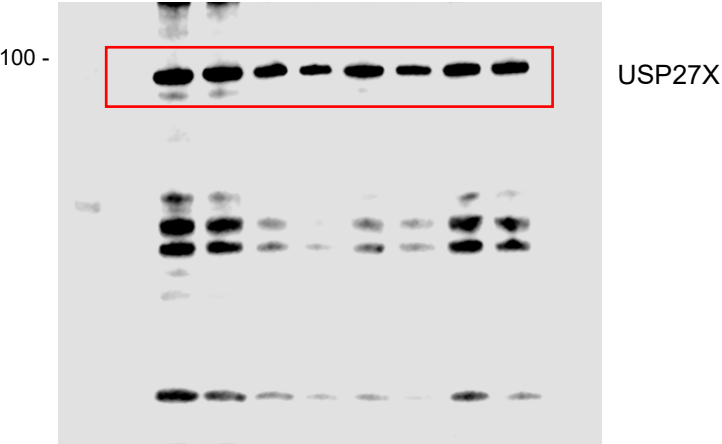

IB: GST (USP27X)

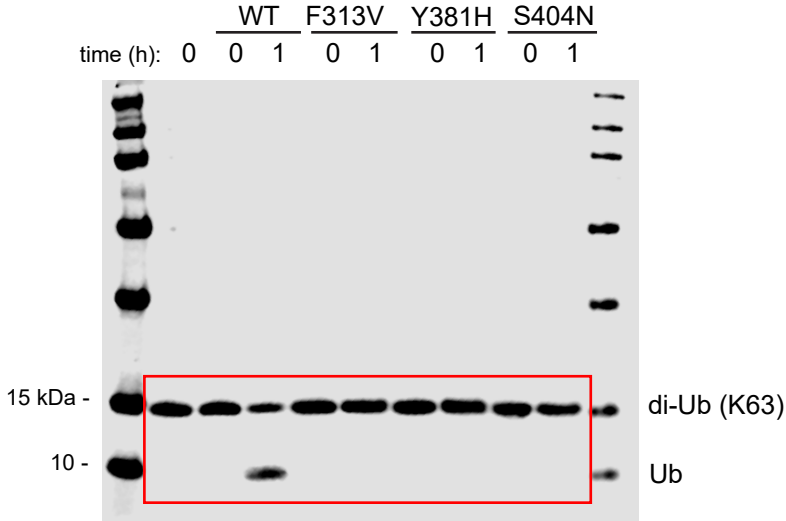

IB: Ubiquitin

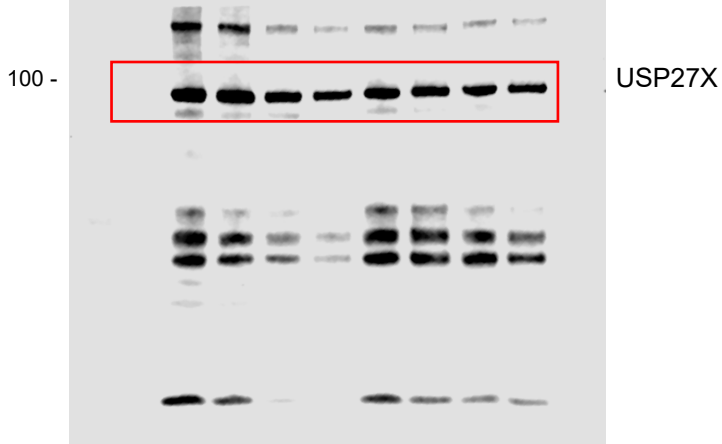

IB: GST (USP27X)

Supplement: Supplementary file 9 [file LSA-2023-02258_SdataF4.2.pdf]

Figure 4C

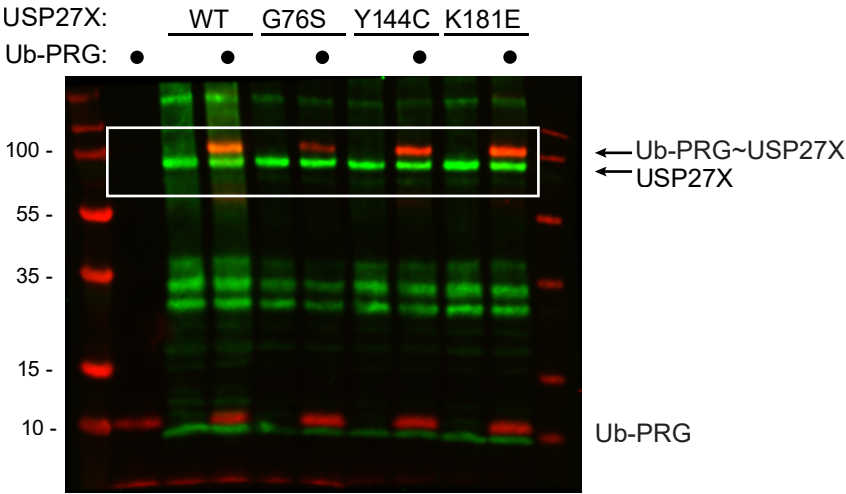

merge: USP27X/Ub-PRG

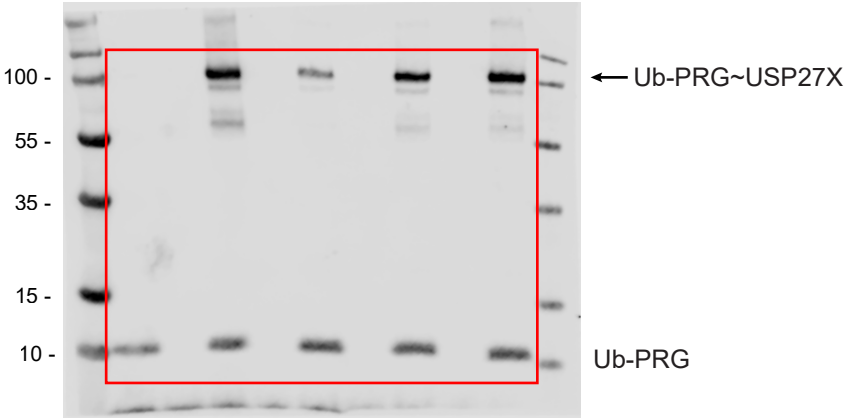

IB: HA (Ub-PRG)

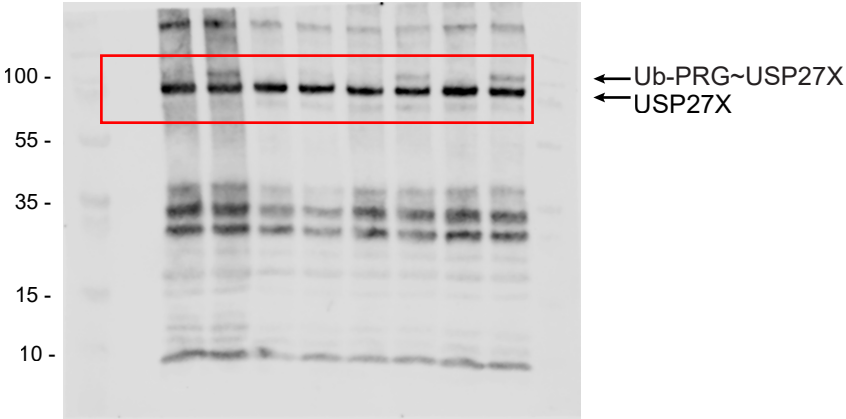

IB: GST (USP27X)

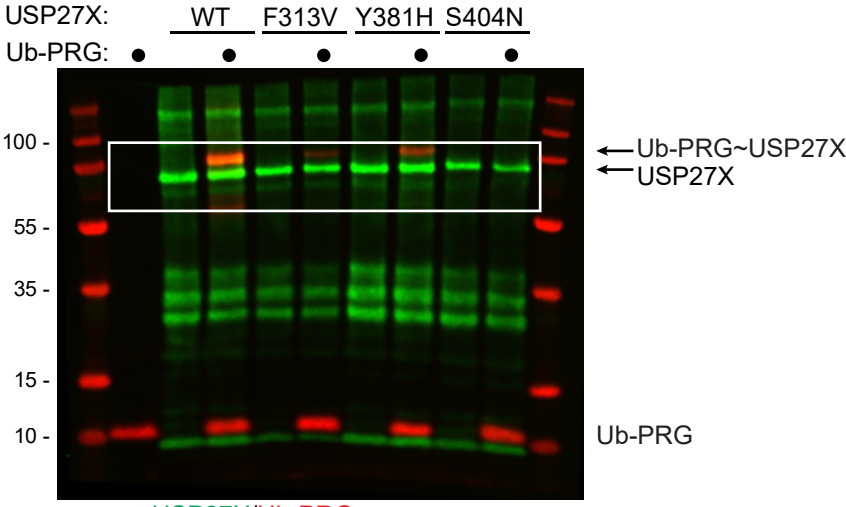

merge: USP27X/Ub-PRG

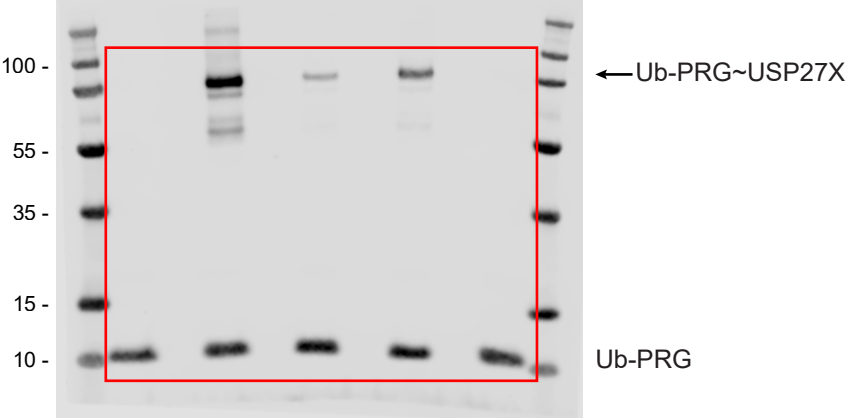

IB: HA (Ub-PRG)

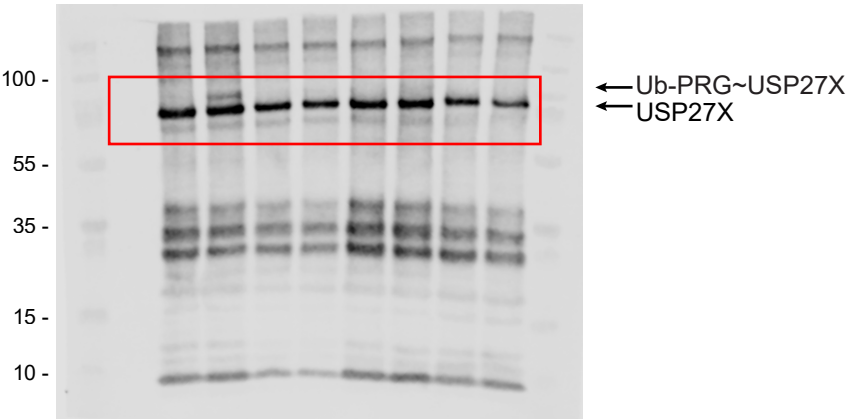

IB: GST (USP27X)

Supplement: Supplementary file 10 [file LSA-2023-02258_SdataF4.3.pdf]

Figure 5A

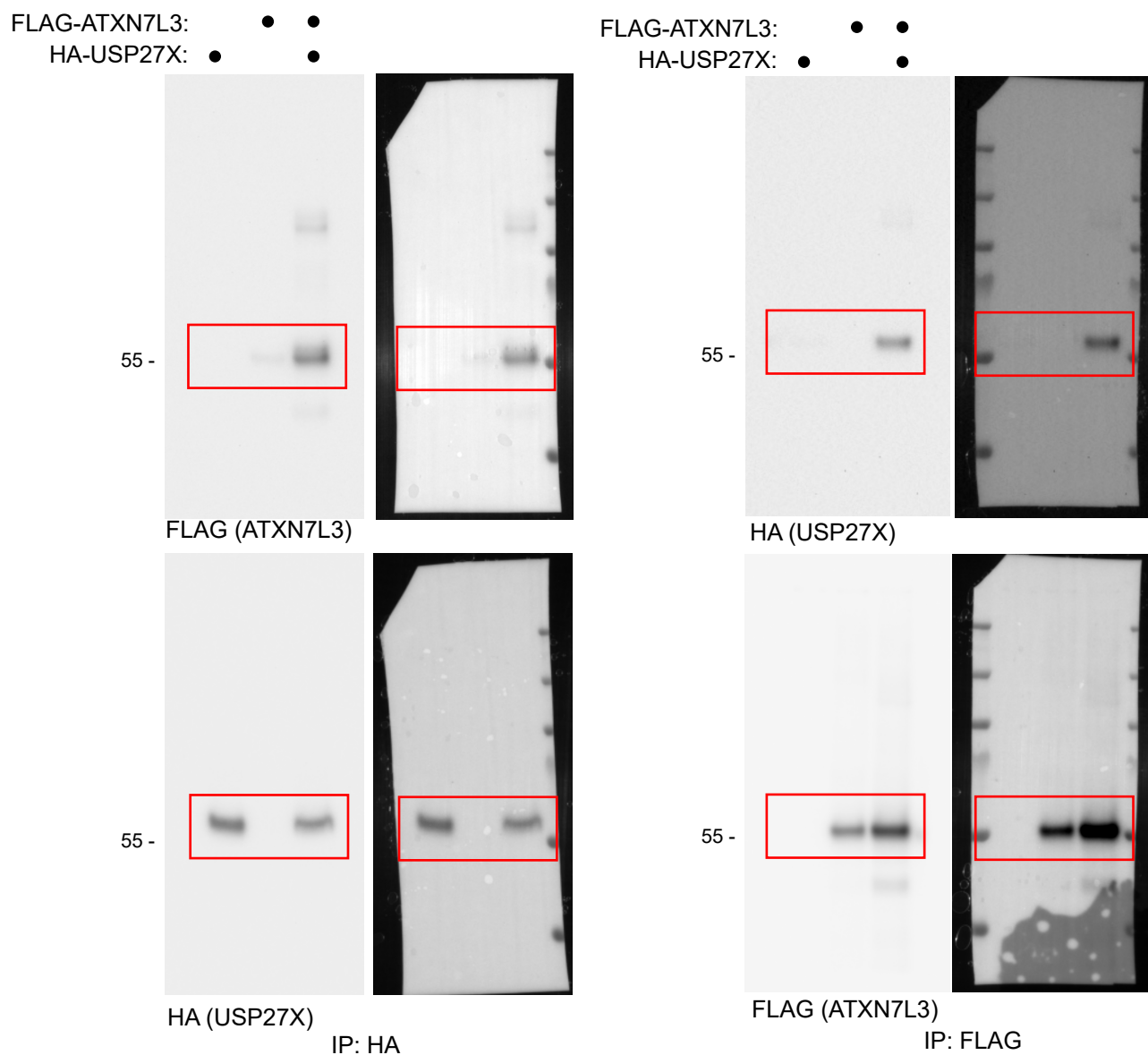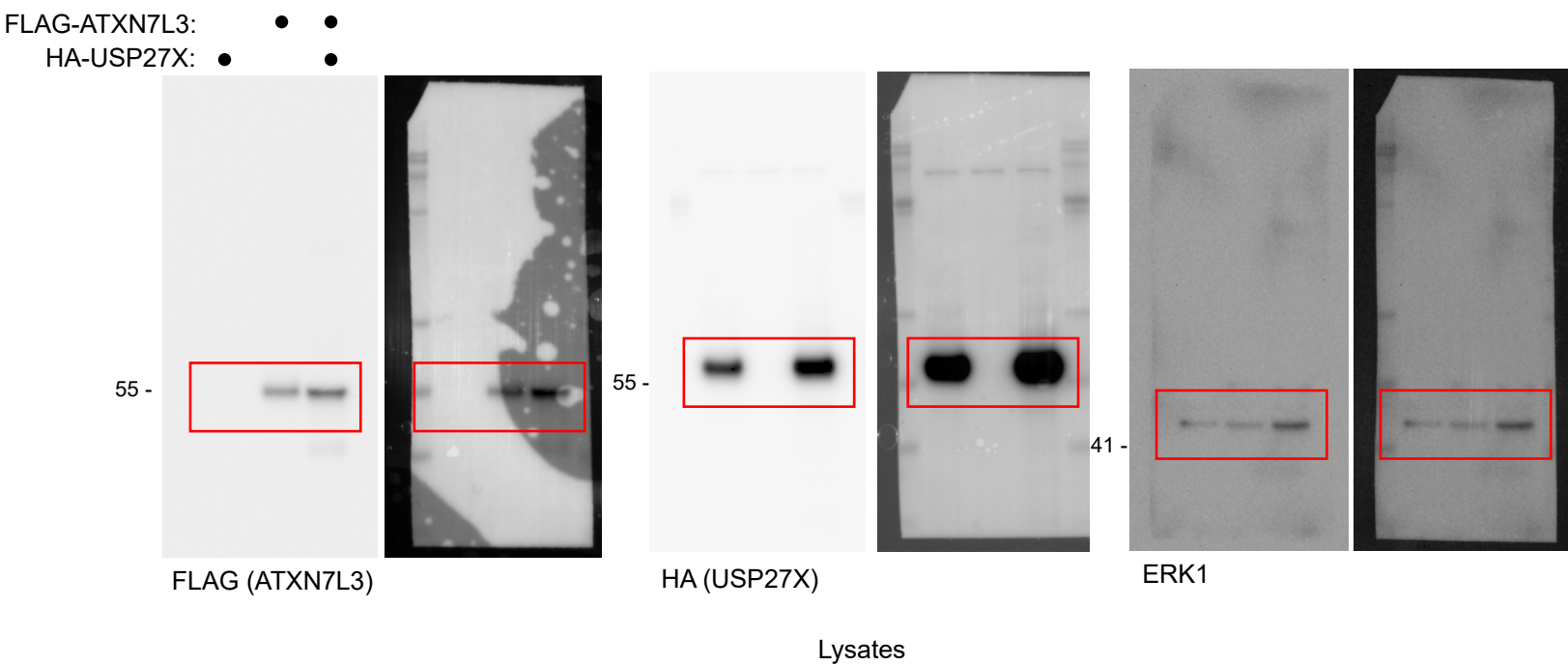

Supplement: Supplementary file 11 [file LSA-2023-02258_SdataF5.1.pdf]

Figure 5B

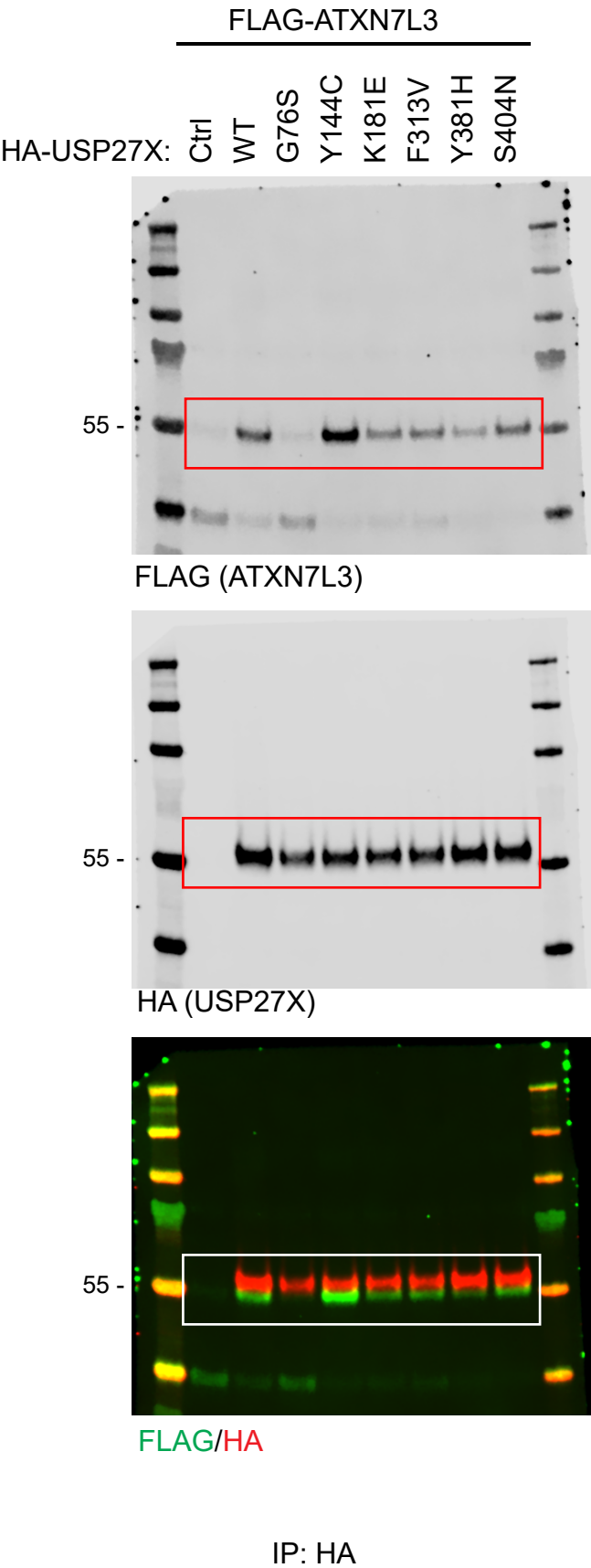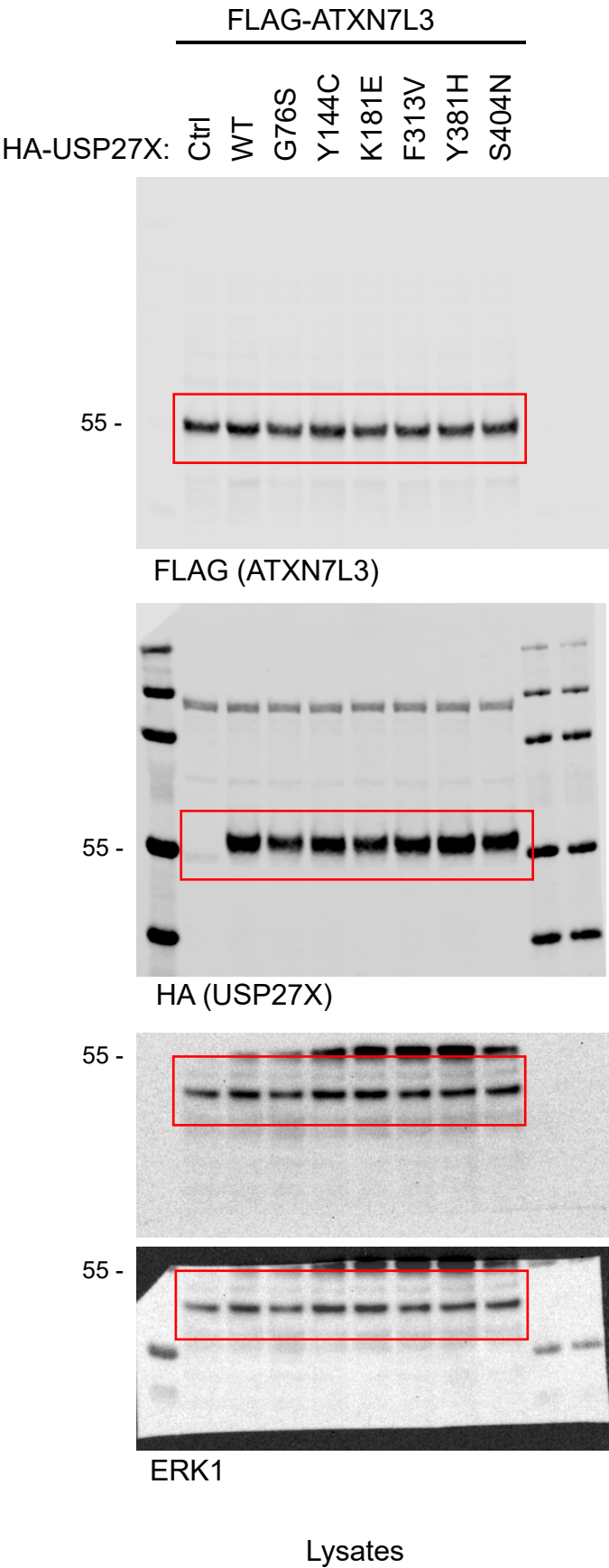

Supplement: Supplementary file 12 [file LSA-2023-02258_SdataF5.2.pdf]
